# Cauda equina syndrome—a practical guide to definition and classification

**DOI:** 10.1007/s00264-021-05273-1

**Published:** 2021-12-04

**Authors:** Chris Lavy, Paul Marks, Katerina Dangas, Nicholas Todd

**Affiliations:** 1 Nuffield Department of Orthopaedics, Rheumatology, and Musculoskeletal Sciences, University of Oxford, Oxford, OX3 7LD UK; 2 Office of Her Majestys’ Coroner, Kingston upon Hull, UK; 3 Newcastle Nuffield Hospital, Newcastle upon Tyne, UK

**Keywords:** Cauda equina syndrome, Definition, Classification, Disc herniation

## Abstract

**Purpose:**

International uniformity of definition and classification are crucial for diagnosis and management of cauda equina syndrome (CES). They are also useful for clinicians when discussing CES with patients and relatives, and for medicolegal purposes.

**Methods:**

We reviewed published literature using PubMed on definition and classification of cauda equina syndrome since 2000 (21 years). Using the search terms ‘cauda equina’ and ‘definition’ or ‘classification’, we found and reviewed 212 papers.

**Results:**

There were 17 different definitions of CES used in the literature. There were three well-defined methods of classification of CES. The two-stage system of incomplete CES (CESI) versus CES with retention (CESR) is the most commonly used classification, and has prognostic value although the details of this continue to be debated.

**Conclusion:**

We used the existing literature to propose a clear definition of CES. We also drew on peer-reviewed published literature that has helped to amplify and expand the CESI/CESR dichotomy, adding categories that are both less severe than CESI, and more severe than CESR, and we propose clear definitions in a table form to assist current and future discussion and management of CES.

## Introduction

**Fig. 1:**
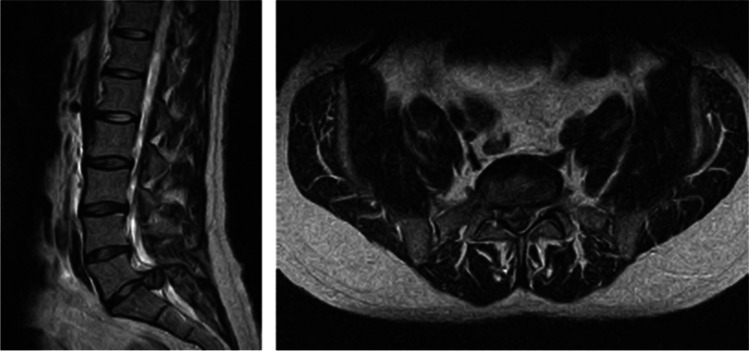
MRI scan example of a disc herniation causing cauda equina compression. The sagittal image on the left shows a large posterior disc herniation at L5/S1 level, and the axial scan on the right at the L5/S1 level shows that the disc herniation fills almost the entire spinal canal, and compresses the cauda equina

Cauda equina syndrome (CES) is a rare but important condition whose commonest cause is massive lumbar disc herniation compressing the roots of the cauda equina in the lower lumbar spinal canal [1] (see Fig. 1). If decompressive surgery is delayed, there can be catastrophic consequences for the patient in terms of bladder, bowel and sexual function. There are also significant legal and financial consequences for treating clinicians and health institutions as litigation for cauda equina syndrome is increasing [2].

There have been encouraging developments in the UK with the publication of guidelines for the management of CES, in particular the standards of care document produced by the British Association of Spine Surgeons [3] which represents all the British Spine Societies, and also the 2018 revision of Red Flag warnings [4] by the National Institute of Clinical Excellence (NICE).

Controversies remain amongst spinal experts in both orthopaedic and neurosurgical specialties over the indications for MRI scanning, the timing of surgery, the nature of surgery and the likely outcomes, and these inevitably form the subject matter for discussions with legal experts. There are several helpful reviews of the published literature on timing and outcome, e.g. [5–9], and although these cover crucial issues in terms of CES management, it is not the purpose of this short paper to consider these.

In order to have constructive conversation with patients and their families, between clinicians, and with legal experts, it is important to have a clear definition of CES, and defined terminology for the stages and types of CES. The purpose of this paper is to review recent literature, to put forward a clear and useful definition of CES, and a clear outline of peer-reviewed clinical classification terminology that will be helpful as a baseline to all parties involved in the management and review of cauda equina cases.

## Methods

Although the relevant literature was well known to the senior authors, to inform this paper a literature review over the last 21 years was performed using PubMed, searching on the terms ‘cauda equina’ and ‘classification’ or ‘definition’. This produced 212 papers all of which were reviewed and analysed to assess what CES definitions and classifications were used. These findings were then discussed by the authors and informed the discussion and recommendations.

## Results and discussion on definition

Anatomically, the term cauda equina comprises all the roots that emerge from the spinal cord where it terminates at around L2. The proximal constituents of the cauda equina therefore comprise components of the L3, L4, L5 and S1 roots. Dysfunction of these roots mainly affects the legs and does not result in what is known as ‘cauda equina syndrome’ which historically is only used to describe the results of dysfunction of the nerve roots below S1, namely S2, S3, S4 and S5.

Fraser and colleagues [10] have identified no less than 17 definitions of CES in the literature. Our search confirmed this. They note that imprecise terminology makes meaningful analysis and comparison between different studies difficult, if not impossible. Taking account of all definitions they have suggested, that for a diagnosis of CES to be made, one or more of the following symptoms or signs must be present:

- Bladder and/or bowel dysfunction
- Reduced sensation in the saddle area
- Sexual dysfunction, with possible neurological deficit in the lower limb (motor/sensory loss, reflex change).

As the commonest cause of CES is lumbar disc herniation, most cases of CES also have back pain and uni- or bilateral sciatica or leg pain, sensory loss and/or weakness. However, these are not essential to the definition.

The diagnosis of CES is initially a clinical one but there are non-CES and non-organic causes of the above symptoms and signs; thus, the clinical diagnosis requires imaging support, usually by an MRI scan. No diagnosis is ever 100% certain and Fairbank et al. [11] remark philosophically that after investigations: “a judgment has to be made if there is cauda equina compression that might be relieved by surgery. Surgical success is if there are no residual symptoms, but there cannot be complete certainty that this would not have happened by natural resolution. Surgical failure is CES.”

## Recommendations on definition

We acknowledge the work of Fraser’s group and agree with their list of key symptoms and signs; therefore, we propose as a practical definition the following:

*Cauda equina syndrome is a clinical diagnosis resulting from dysfunction of one or more of the sacral nerve roots S2 and below. One or more of the following symptoms or signs must be present:*

- *Bladder and/or bowel dysfunction*
- *Reduced sensation in the saddle area*
- *Sexual dysfunction*

*Back and leg pain, and lower limb motor or sensory changes are often present but are not essential to the diagnosis.*

*Nerve root compression is the commonest cause and MRI scanning is usually needed for confirmation.*

## Results and discussion on classification

Before discussing classification, we should note that any classification system needs to use accurate information about the patients’ symptoms and clinical findings regarding bladder, bowel, perineal and genital function and sensation. It is not the place of this paper to go into detail of how these are obtained but it is the observation of the senior authors that on reviewing records of cases of CES, it is common to find that insufficient detail is recorded about bladder and micturition symptoms. It is inappropriate to simply ask if the patient is incontinent. Incontinence is a late stage of CES, and in most cases before incontinence is reached there are subtle changes of urinary sensation, flow and frequency that must be enquired about. Similarly, both subjective and objective features of perineal, perianal and genital sensation need to be assessed.

### Classification by presentation

Tandon and Sankaran [12] classified CES into three types on the basis of presentation.

- Type 1 A rapid onset of CES symptoms with no history of back problems.
- Type 2 Acute bladder/CES symptoms with a history of back problems and sciatica
- Type 3 Longstanding back problems and gradually progressive CES often with spinal stenosis

Those who deal with CES on a regular basis recognise these presentations although the classification system itself is not well known or used, and does not inform treatment. From a litigation point of view, the vast majority of patients are in type 2. Type 1 is rare, and type 3 is usually non-urgent, and occurs in a significantly older age group that is more tolerant of some reduction in bladder and bowel function and is less likely to be sexually active.

### Multifactorial classification

Shi and co-workers [13] proposed a complex multifactorial classification that has not been widely adopted or tested. They proposed four groups:

- Group 1 (preclinical) was defined by low-back pain with only bulbocavernosus reflex (BCR) and ischiocavernosus reflex (ICR) abnormalities and no typical symptoms of CES.
- Group 2 (early) had saddle sensory disturbance, numbness and bilateral sciatica.
- Group 3 (middle) had saddle sensory disturbance, numbness, bowel and/or bladder dysfunction, motor weakness of the lower extremities and reduced sexual function.
- Group 4 (late) had complete absence of saddle sensation and sexual function, and uncontrolled bowel function.

This is an interesting classification; however, it has limitations; for example, in group 1, very few patients would consent to bulbocavernosus reflex testing, when all they had was back pain. In addition, few clinicians or researchers use this classification and we will not discuss it further.

### Scan-negative cauda equina syndrome

Hoeritzauer and colleagues [14] introduce the concept of ‘scan negative CES’ where the patient has CES-type symptoms however CES compression is not the cause as by definition there are no compressive features on the MRI scan. This group is clearly non-surgical and although interesting will not be further discussed here.

### CESI/CESR classification

The most commonly used classification in surgical or compressive CES is the binary division between incomplete CES (CESI) and CES with painless bladder retention (CESR).

This is well defined by Gleave and Macfarlane in their 2002 paper [15]. They define CESI as CES where there are urinary difficulties of neurogenic origin such as altered urinary sensation, loss of desire to void, poor stream or the need to strain, but there is still executive control of bladder function and voiding is possible even if difficult. CESR occurs when the bladder is no longer under executive control and there is painless retention of urine with overflow. We note that the diagnosis of CESR is made more complex when early catheterisation is performed, as bladder distension and overflow are prevented.

This CESI/CESR classification is useful as it reflects the degree of damage to the sensitive cauda equina nerves and therefore is of prognostic value for future function. Gleave and Macfarlane also claim that it has value in planning management because they suggest that a die is cast, when a patient reaches CESR. “Once CESR had occurred, the timing of operation had no influence on outcome.” and “it must be concluded that the outcome of CESR has already been decided by the time the patient has been admitted to hospital.” There are those who dispute this, e.g. De Long [16], but nevertheless the classification system is well accepted.

Some authors state that in order to have a CESI diagnosis, the patient also needs to have altered perineal sensation. This was not a criterion mentioned by Gleave and Macfarlane in their 2002 definition. However, it is implied in their definition because it underpins the neurogenic origin of bladder problems; i.e., in the absence of impaired perineal sensation, bladder problems are unlikely to be neurogenic (perhaps due to pain etc.). Our view is that although reduced perineal sensation is commonly present with CESI, we agree with Gleave and Macfarlane that it is not absolutely mandatory to make the diagnosis. This view is supported by Barraclough [17] who in a short review of relevant literature reports many cases of MRI-confirmed CES where there is no loss of perineal sensation.

Our review of the literature revealed several subsequent additions of further categories to CESI/CESR.

#### CESE

Todd has suggested a category of early CES (CESE) where there is reduced perineal sensation but normal bladder function, or a change in micturition pattern with normal perineal sensation [18]. In our opinion, CESE is a useful concept and alerts attention to cases that might progress to CESI. CESE is better called ‘symptom-only CES’ which is a term that is easy to understand and communicate. It is also a stage where there is likely to be an excellent outcome to decompression as the condition is so early.

#### CESS

In the 2009 guidelines published by the Society of British Neurosurgeons (SBNS) [19], a pre-CES category is noted and called ‘suspected CES’ (CESS). It is not clearly defined and the guidelines simply state:

“*Suspected cauda equina syndrome (CESS) cases of severe back and leg pains with variable neurological symptoms and signs, and a suggestion of sphincter disturbance*”.

Todd in his 2017 paper [18] refines CESS as occurring in cases where there are no actual CES symptoms, but only bilateral leg symptoms, which raise suspicion of a large disc herniation simultaneously compressing roots on both sides of the spinal canal. To this category could also be added patients in whom previous MRI scans have shown a pre-existing large disc herniation although there are no actual CES symptoms. We propose therefore that the category CESS encompasses both clinical and radiological subgroups. In both, there are as yet no CES symptoms or signs, but the potential for them to occur is present.

#### CESC

In addition to CESE and CESS where there are earlier or milder symptoms than CESI, many clinicians describe CESC (complete CES) where there is loss of all CES sensory and motor function. We have been unable to find the specific origin of this term. This is clearly a worse clinical situation than CESR and serves to remind those managing CES cases that although in CESR there is loss of executive bladder control with overflow incontinence, there can be preservation of some useful perineal and genital/sexual sensation, and preservation of bowel function, and delay in decompression may result in loss of these, leading to a worse situation than CESR. Thus although as Gleave and Macfarlane maintain, in moving from CESI to CESR a rubicon has been crossed, and recovery of normal function is less likely, all is not lost, there is ‘further to fall’, and unrelieved cauda equina compression in CESR can lead to a much worse situation.

## Recommendations on classification

We acknowledge the key work of Gleave and Macfarlane, and the key place of the CESI/CESR dichotomy in classifying CES, but with the addition of the three further categories discussed above, we propose the following comprehensive classification (Table 1).

**Table 1:** Comprehensive classification of cauda equina syndrome

Name	Abbreviation	Definition
Suspected CES	CESS	No bladder/bowel/genital/perineal symptoms, but bilateral sciatica or motor/sensory loss in legs. (this is clinical CESS) Or known large disc herniation on existing MRI (this is radiological CESS)
Symptom-only CES (early CES)	CESE	Normal bladder, bowel and sexual function but some sensory loss in perineum or change in micturition frequency
Incomplete CES	CESI	Alteration in bladder/urethral sensation or function, but maintenance of executive bladder control. + / − perineal sensory changes, or sexual or bowel sensory or functional changes
CES with retention	CESR	As in 3 but with painless bladder retention and overflow
Complete CES	CESC	Insensate bladder with overflow incontinence, no perineal perianal or sexual sensation, no anal tone

## Data Availability

There is no data store for this paper.
